# Dendriplex-Impregnated Hydrogels With Programmed Release Rate

**DOI:** 10.3389/fchem.2021.780608

**Published:** 2022-01-05

**Authors:** Evgeny Apartsin, Alya Venyaminova, Jean-Pierre Majoral, Anne-Marie Caminade

**Affiliations:** ^1^ Laboratoire de Chimie de Coordination, CNRS, Toulouse, France; ^2^ LCC-CNRS, Université de Toulouse, CNRS, Toulouse, France; ^3^ Institute of Chemical Biology and Fundamental Medicine SB RAS, Novosibirsk, Russia; ^4^ Novosibirsk State University, Novosibirsk, Russia

**Keywords:** dendrimers, oligonucleotides, hydrogel, polyelectrolyte complexes, controlled release

## Abstract

Hydrogels are biocompatible matrices for local delivery of nucleic acids; however, functional dopants are required to provide efficient delivery into cells. In particular, dendrimers, known as robust nucleic acid carriers, can be used as dopants. Herein, we report the first example of impregnating neutral hydrogels with siRNA–dendrimer complexes. The surface chemistry of dendrimers allows adjusting the release rate of siRNA-containing complexes. This methodology can bring new materials for biomedical applications.

## Introduction

Local delivery of therapeutic nucleic acids, alone (Sarett et al., 2015) or in combination with other drugs (Larsson et al., 2017), is an emerging topic in nanomedicine. To date, various approaches have been developed to deliver nucleic acid constructions locally, either into the skin (Rogers et al., 2013; Vij et al., 2017) or internal organs (Kwekkeboom et al., 2015; Xie et al., 2020). Depending on the application, a long-term treatment may be required. Therefore, materials are needed to provide a sustained drug release into tissues in contact. For instance, hydrogels are convenient matrices for local drug delivery, in particular for the delivery of therapeutic nucleic acids (Fliervoet et al., 2018). They are also frequently combined with hard or soft nanoparticles complexing nucleic acids and acting as carriers at the cellular or tissue level (Fattal et al., 2004; Wang and Burdick, 2017; Saleh et al., 2019). The choice of a carrier system is crucial as it can define the therapeutic performance of a material as well as modulate its physicochemical properties. Dendritic molecules, that is, dendrimers and dendrons, can be well suited for this purpose (Caminade, 2016; Caminade, 2017; Apartsin and Caminade, 2021).

Dendritic molecules are hyperbranched macromolecules of precisely defined molecular structure exposing numerous functional groups on the periphery. Due to the richness of the surface chemistry, dendrimers and dendrons can be functionalized with biomimetic moieties and therefore used as biocompatible carriers for both low-molecular drugs (such as anticancer chemodrugs) and macromolecular therapeutic substances (DNA, mRNA, proteins) (Hsu et al., 2017; Knauer et al., 2019; Mignani et al., 2020). Decorating the surface with cationic moieties promotes the interaction of dendrimer-based complexes (dendriplexes) or supramolecular assemblies with the cell surface inducing endocytosis. Inherent dendrimer multivalency, together with precise structure, is their advantage over other classes of macromolecular carriers. Having been applied to the nucleic acid delivery, dendrimers have shown considerable binding capacity and high efficiency for the internalization into target cells, inducing programmed therapeutic effects *in vitro* and *in vivo* (Palmerston Mendes et al., 2017; Dzmitruk et al., 2018). In particular, topical delivery of therapeutic nucleic acids into skin tissue can be achieved. Recent findings show that dendrimer-assisted topical delivery can be accomplished even for thousand-base-long self-amplifying RNA (Saviano et al., 2020), opening new opportunities for dendrimers in nanomedicine.

Due to the presence of multiple functional groups on the surface, dendrimers can be used as cross-linking moieties to develop dense hydrogel networks (Nummelin et al., 2015; Hodgson et al., 2017; Wang et al., 2017). Dendrimer-containing hydrogels can be loaded with bioactive compounds and show good therapeutic activity upon topical application (Conde et al., 2016; Wang et al., 2016; Xu et al., 2017). These hydrogels have been shown to possess functional performance similar to commercially available hydrogel species (Villa-Camacho et al., 2015).

The synergistic combination of two methodologies, namely, dendrimer-mediated nucleic acid delivery and hydrogel-based local drug delivery, can yield highly biocompatible materials for the long-term local delivery of therapeutic nucleic acids into target tissues. Conceptually, the hydrogel scaffold is to bring biocompatibility or bioresorption and to regulate the rate of drug release, whereas the dendrimer is to provide highly efficient and specific delivery of nucleic acid therapeutics into cells that are in contact with a biomaterial. However, no such system has been reported yet.

Herein, we report a proof-of-concept study in preparing neutral hydrogel scaffolds impregnated with nanoscale polyelectrolyte complexes of therapeutic nucleic acids and polycationic dendrimers. We hypothesized that the entrapment of complexes into a hydrogel network will result in their long-term release. That would be highly useful for the design of biomaterials for local drug delivery.

## Materials and Methods

Polycationic phosphorus dendrimers were synthesized according to previously published procedures (Ihnatsyeu-Kachan et al., 2017; Apartsin et al., 2018). Mcl-1 siRNA (sense strand: 5′-GGACUUUUAUACCUGUUAUtt-3′-FAM; antisense strand: 5′-AUA​ACA​GGU​AUA​AAA​GUC​Ctg; lowercase letters denote deoxyribonucleotides) was synthesized and annealed as described in Krasheninina et al. (2019).

### Dendriplexes Formation

Dendriplexes were formed by siRNA and dendrimers in an RNase-free PBS buffer (10 mM phosphate buffer, pH 7.4, 137 mM NaCl, 2.7 mM KCl), followed by incubation for 10 min at 25°C. The dendrimer-to-siRNA charge ratio (i.e., the excess of cations over anions) was calculated as follows:

$$CR=\frac{N_{+}C_{D}}{N_{-}C_{siRNA}},$$

where CR is the charge ratio; $N_{+}=48$ is the number of cations per dendrimer molecule; $N_{-}=40$ is the number of anions per siRNA molecule; $C_{D}$ is the dendrimer concentration; and $C_{siRNA}$ is the siRNA concentration in a sample.

### Gel Retardation Assay

The ability of the cationic dendrimers to form complexes with siRNAs was studied by gel electrophoresis in 1% agarose gel. Dendriplexes were prepared by mixing siRNA (40 pmol per sample), ethidium bromide (EB) (0.4 µM, ∼1 EB molecule per 2 bp of siRNA), and dendrimers (at increasing concentrations depending on the charge ratios) and dissolved in PBS. After 15-min incubation at 25°C, electrophoresis was carried out in 1% agarose gel at 80 V (Mini-Sub^®^ Cell GT, Bio-Rad, United States) in TBE buffer (89 mM Tris–borate, pH 8.4, 10 mM Na_2_EDTA), and the bands were visualized under a UV using gel documentation system (Helicon, Russia).

### Fluorescence Polarization

Mcl-1 siRNA (1 µM) in PBS (30 µL) was placed in wells of a black Costar 96 half-area microplate (Costar, United States). A solution of dendrimer AG3, TG3, or PG3 was added gradually to achieve the desired charge ratio from 0.25 to 5. In a control experiment, water was added, instead of dendrimer solution. After each addition, solutions were mixed by pipetting and incubated for 5 min; then fluorescence polarization values were read using a microplate reader (BMG Labtech, Germany). The experiments were performed in triplicate, and results were presented as mean ± S.D.

### Atomic Force Microscopy

An aliquot of dendriplex solution was dropped on a mica slide for 1–2 min. The slide was then washed 3 times with deionized water and air-dried. Scanning was performed in the tapping mode using a Multimode 8 atomic force microscope (Bruker) with NSG10_DLC cantilevers with a tip curvature radius of 1–3 nm (NT-MDT, Russia) at a scanning rate of 3 Hz. Images were processed using Gwyddion 2.36 software.

### Hydrogel Impregnation and Dendriplex Release

Dendriplexes were formed by mixing Mcl-1 siRNA (100 µM) and dendrimers (AG3, TG3, PG3 or mixtures AG3/TG3, AG3/PG3) at dendrimer-to-siRNA charge ratio of 5 in 15 µL PBS, followed by incubation for 15 min at 25°C. Then, 10 µL of a dendriplex solution was added to 40 µL of hot 2% agarose solution in PBS. When the solution cooled down to room temperature and hydrogel was formed, 100 µL of PBS was added, and the gel was gently shaken at 25°C. 5 µL aliquots were taken at 0, 10, 20, 30 min, 1, 2, 3, 16, and 24 h of incubation; diluted in 30 µL of PBS; and transferred into wells of a black Costar 96 half-area microplate (Costar, United States); fluorescence intensity and fluorescence polarization values were read using a microplate reader (BMG Labtech, Germany). The remaining 5 µL dendriplex solution was treated in the same way and used as a control. The experiments were performed in triplicate, and the results were represented as mean ± S.D. To fit release values in kinetic profiles, the exponential model was used. Fitting was considered satisfactory if *r* ^2^ > 0.95.

## Results and Discussion

Choosing a matrix for a hydrogel network, we were looking for a neutral compound, either biomimetic or of biological origin, able to form hydrogels with pores of 100–200 nm diameter (comparable to sizes of polyelectrolyte complexes). For instance, agarose is a good candidate, for it is a cheap mass produced biocompatible polymer forming soft bioresorbable physical hydrogels (Zarrintaj et al., 2018). The mean pore diameter in agarose hydrogels can be simply controlled by the agarose percentage (Pluen et al., 1999; Narayanan et al., 2006).

As a bioactive cargo, we have chosen small interfering RNA (siRNA) Mcl-1 possessing anticancer activity. This siRNA activates programmed cell death by suppressing the expression of one of the antiapoptotic proteins of the Bcl-2 family regulating the mitochondrial apoptosis pathway (Chetoui et al., 2008; Guoan et al., 2010; Krasheninina et al., 2019).

**FIGURE 1:**
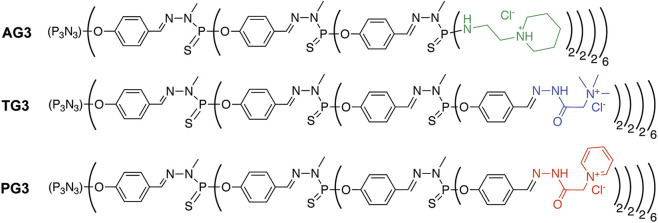
Structures of polycationic phosphorus dendrimers used for preparing dendriplexes.

As carriers, we have chosen phosphorus dendrimers bearing cationic groups on the periphery. Phosphorus dendrimers are widely used as nanodrugs *per se* (Hayder et al., 2011; Caminade et al., 2015) and as carriers for low-molecular and macromolecular bioactive compounds. For instance, polycationic phosphorus dendrimers of high generations are versatile carriers for intracellular delivery of nucleic acid constructions such as siRNA (Ferenc et al., 2013; Ionov et al., 2015; Dzmitruk et al., 2015; Bohr et al., 2017; Deriu et al., 2018; Ihnatsyeu-Kachan et al., 2017) or plasmid DNA (Loup et al., 1999; Padié et al., 2009). Herein, we have used three types of dendrimers of generation 3 bearing 48 surface groups each: piperidinium chloride (AG3), trimethylammonium chloride acetohydrazone (Girard reagent T; TG3), and pyridinium chloride acetohydrazone (Girard reagent P; PG3). The structures of dendrimers are given in Figure 1. Dendrimers TG3 and PG3 can form hydrogels through multiple hydrogen bonds between branches, with biomimetic additives facilitating the gelation (Marmillon et al., 2001; Apartsin et al., 2018). Such dendrimer hydrogels were able to bind oligonucleotides reversibly. Dendrimer AG3, though unable to form hydrogels, has been shown to provide highly efficient delivery of anticancer siRNAs into tumor cells inducing apoptosis (Ihnatsyeu-Kachan et al., 2017).

As dendrimers bind siRNAs by means of electrostatic interactions, the dendrimer-to-siRNA charge ratio strongly matters. To find a ratio, where siRNA is mostly bound, we mixed it with three cationic dendrimers at different ratios and analyzed the complexes formed by means of agarose gel electrophoresis upon visualization with ethidium bromide (Supplementary Figure S1).We observed no band of free siRNA at the cation excess >3 for AG3, and at the cation excess >2 for PG3 and TG3. This difference can be explained by the presence of hydrophilic hydrazone moieties on the surface of PG3 and TG3 dendrimers, which makes peripheral cations more available to the complexation with oligonucleotides. To evaluate how strongly dendrimers bind siRNA, we measured the fluorescence polarization of 3′-fluorescein–labeled siRNA upon complexing with AG3, PG3, and TG3. Fluorescence polarization assay is sensitive to hindering of the fluorophore rotation in an oligonucleotide upon complexation and thus gives information about the strength of the siRNA complexation. The fluorescence polarization values grew upon the cation excess reaching a plateau at the ratio > 3 (Supplementary Figure S2) as it is supposed to (Szewczyk et al., 2012; Conti et al., 2014). However, in the case of AG3- and TG3-containing complexes, the siRNA binding leads to the ∼2.8-fold increase in polarization, whereas in PG3-containing complexes, >6-fold increase was observed. The most likely reason for such a difference is the difference in the geometry of peripheral cations and their availability for the interaction with the sugar–phosphate backbone (Deriu et al., 2018). Furthermore, hydrophobic interactions between siRNA and dendrimers should be taken into account, for they are known to contribute to the complexation along with electrostatic interactions (Slita et al., 2007; Filippov et al., 2010). To work with dendriplexes, where siRNA is fully saturated with dendrimers, we have used the 5-fold cation excess for further experiments.

**FIGURE 2:**
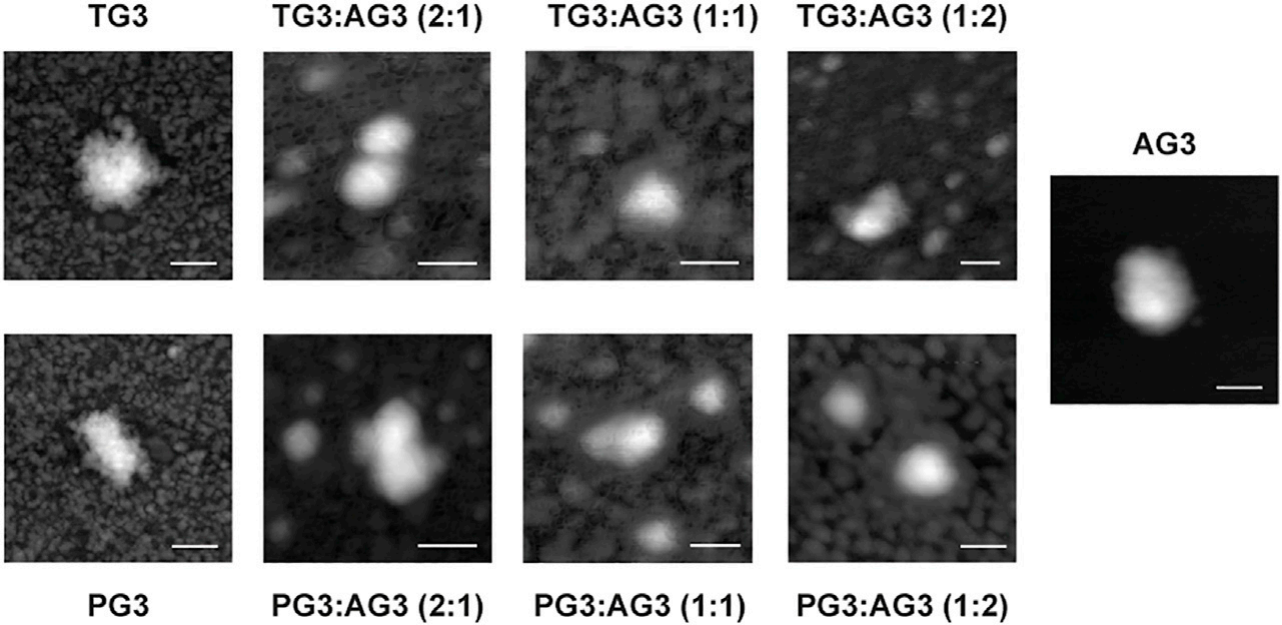
Representative AFM images of dendriplexes. Charge ratio 5. Scale bar is 100 nm.

To estimate the size of dendriplexes, we did AFM of samples just after adsorption on a mica slide. This allowed us to visualize dendriplexes, though not in their native form as in solution, but not dehydrated either. Observed particles were round, and their mean size was 100 nm (Figure 2). The composition of dendriplexes did not significantly change their size, even when mixtures of dendrimers at different proportions were used to form dendriplexes (see below). Size values obtained by DLS measurements (Supplementary Figure S3) were in good agreement with the AFM data.

**FIGURE 3:**
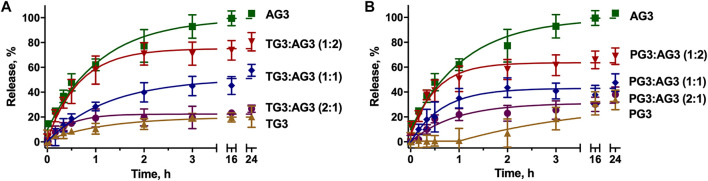
Kinetic profiles of dendriplexes release from the agarose gel. Dendriplexes contain either AG3 and TG3 **(A)** or AG3 and PG3 **(B)** in different ratios. Charge ratio 5.

**FIGURE 4:**
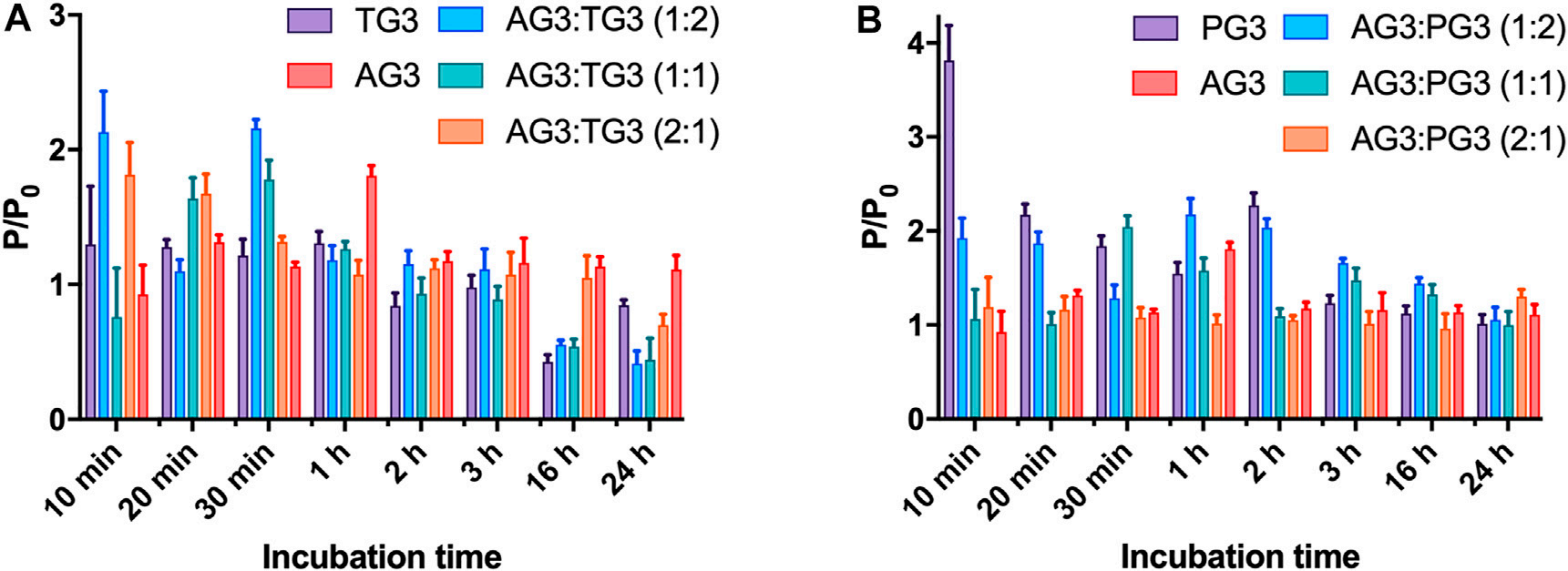
Evolution of fluorescence polarization of siRNA upon release of dendriplexes from the agarose gel. Dendriplexes contain either AG3 and TG3 **(A)** or AG3 and PG3 **(B)**. The ratio *P/P*_*0*_ represents the ratio of fluorescence polarization values in a sample (*P*) and that of free siRNA (*P*_*0*_). Charge ratio 5.

To impregnate dendriplexes into a hydrogel network, we added them to hot 2% agarose solution prior to gelation. Cooling down, agarose forms a physical gel entrapping dendriplexes in its pores. We then incubated dendriplex-containing hydrogels in a buffer following the release of siRNA by measuring the fluorescence acquisition in eluates. We considered the size of dendriplexes to be smaller than the pore diameter in 1% agarose hydrogel network [100–350 nm (Viovy, 2000; Stellwagen, 2009)], so dendriplexes cannot be physically retained in a hydrogel. We therefore expected that the release rate would be defined mostly by the diffusion of complexes from the hydrogel. Dendriplexes containing AG3 behaved exactly as we predicted: burst release was observed with >90% release being achieved in 3 h. However, surprisingly, dendriplexes containing TG3 and PG3 were released quite poorly; the 50% release was not achieved even after 24-h incubation (Figure 3). We hypothesized that such effects arise from the differences in the chemical structure of dendrimer periphery as apart from that, the dendriplexes were identical. Indeed, dendrimers TG3 and PG3 expose multiple hydrazone moieties on the periphery that can form hydrogen bonds with the hydrogel scaffold. This is a likely reason why these dendriplexes are retained in a hydrogel. With this in mind, we have impregnated hydrogels with dendriplexes containing mixtures AG3:TG3 and AG3:PG3 and studied the siRNA release (Figure 3). We have found that the ratio AG3/TG3 or AG3/PG3 in a dendriplex strongly affects both the rate and completeness of release. The increase in the hydrazone-terminated dendrimer content up to 1/3 does not affect the release rate within the first hour of incubation; however, the increase in the content up to 50% slows the release within the whole 24-h observation span. Interestingly, the profile of release of the PG3-containing dendriplex has a considerable delay in the first hour of incubation. Given that the TG3 dendriplex does not exhibit this delay, we suppose that this phenomenon originates rather from differences in the character of siRNA complexation between TG3 and PG3, as observed in the fluorescence polarization profiles (see above).Whereas fluorescence intensity in samples (Figure 3) represents the overall release of siRNA from the hydrogel, fluorescence polarization (Figure 4) shows the degree of siRNA complexation: the higher the polarization value, the stronger is the siRNA bound to dendriplexes. Basing on the evolution of the fluorescence polarization in samples over time, we suggest that siRNA is released from the hydrogel in the form of dendriplexes. The effects of PG3-containing dendriplexes are more pronounced in comparison with TG3 ones, which agrees with the strength of siRNA complexation by these dendrimers (Supplementary Figure S2). This finding is important as complexation with dendrimers is known to stimulate the cellular uptake of oligonucleotides. Given that endocytosis is quite a quick process, with multiple endocytosis events occurring in a cell in a few-minutes span (Liang et al., 2017), we can assume that released dendriplexes could be endocytosed shortly after release from a hydrogel, before they could decompose.

Thus, varying the content of dendrimers in complexes, we can modulate the speed of their elution from hydrogels. This feature can be used for the precise design of a material for a given biomedical task. For instance, agarose gels are considered prospective biomaterials for the regeneration of cartilage and brain implants (Lecomte et al., 2018; Choi et al., 2020; Salati et al., 2020). Being in contact with tissues, agarose hydrogels undergo bioresorption (Rousselle et al., 2019). This would lead to the degradation of the hydrogel network, driving the release of dendriplexes still retained in a gel network, as it has been shown for cationic silica nanoparticles (Wang et al., 2015). This option can be useful for the sustained release of small quantities of regulatory nucleic acids (siRNA or microRNA). For instance, this technique can be used to achieve long-term local tumor treatment (Han et al., 2011), to improve wound healing (Saleh et al., 2019; Berger et al., 2021), or to suppress local inflammatory reaction (Zhou et al., 2018), which can occur in the proximity of an implant.

## Conclusion

In summary, we have reported the first example of a neutral biocompatible agarose hydrogel impregnated with polyelectrolyte complexes of siRNA with polycationic phosphorus dendrimers. The anchoring of complexes in a gel is due to the formation of numerous hydrogen bonds between cationic moieties on the periphery of dendrimers and the hydrogel scaffold. Changing the content of different dendrimer species in complexes, we have found an easy way to control the rate of release of complexes from a hydrogel. We believe this methodology can be useful for the development of functional hydrogels as local drug delivery systems and tissue engineering tools.

## Data Availability

The raw data supporting the conclusions of this article will be made available by the authors, without undue reservation.
