# Supplementary material for: Dendriplex-Impregnated Hydrogels With Programmed Release Rate
Source: Front Chem. 2022 Jan 5;9:780608. doi: 10.3389/fchem.2021.780608 (PMC8766751; doi:10.3389/fchem.2021.780608)
Supplement: Supplementary file 1 [file DataSheet1.PDF]

Supplementary Material

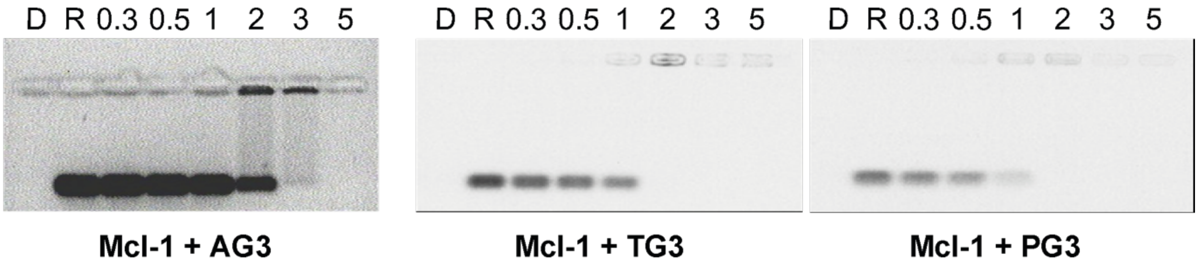

Supplementary Figure 1. Representative 1% agarose gel electrophoregrams of Mcl-1 siRNA complexed with polycationic phosphorus dendrimers. Charge ratio is indicated above. Free dendrimer was added on the line D, free siRNA was added on the line R.

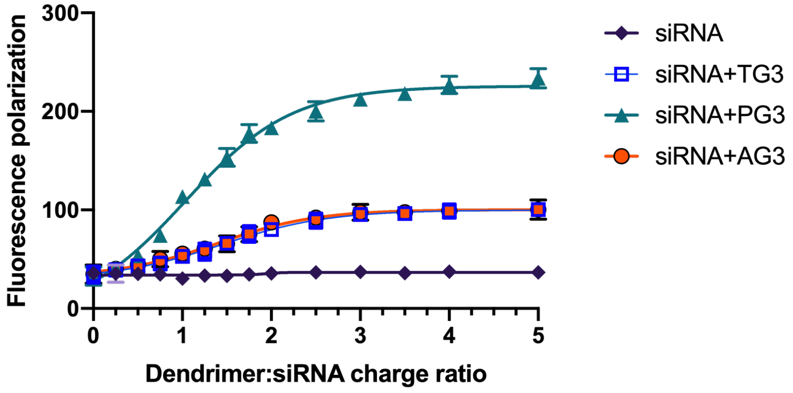

Supplementary Figure 2. Fluorescence polarization profiles upon complexation of Mcl-1 siRNA with dendrimers.

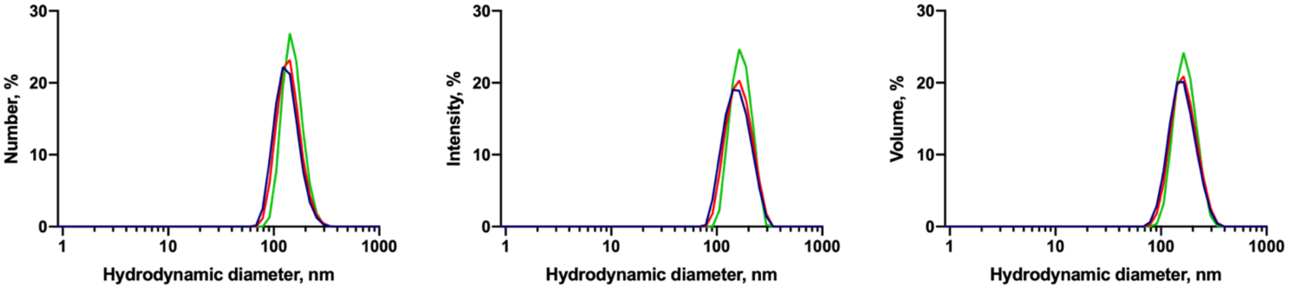

Supplementary Figure 3. Representative DLS profiles of a siRNA+AG3 dendriplex (Zetasizer Nano ZS particle analyzer). siRNA concentration is 250 nM, charge ratio is 5. Data from triplicate measurements are shown.
